# Models

**Published:** 1901-04-15

**Authors:** E. C. Moore


THE
### DENTAL REGISTER.

**Vol. LV.** **April 15, 1901.** **No. 4.**

### MODELS.

BY E. C. MOORE, D.D.S.

Read at a Meeting of the Detroit Dental Society, March 4, 1901.

The subject of models involves the entire field of dental prosthesis. But I shall confine my remarks to the production of suitable models from which metal dies for swaging may be made.

The very name model implies, in degree at least, perfection. A model man is one who is beyond criticism. A model for a bronze or marble statue is valued artistically as it is perfect. The perfection of the foundryman's model determines the value of the casting made from it, and the same in principle obtains in dental models. If the model is imperfect or false the resultant denture is bound to be unsatisfactory. To properly apprehend our subject we must go back to the patient, or at least to the impression, to secure a proper start. How can we secure a perfect model from an imperfect impression? I shall not consume time in discussing the subject of taking impressions, except to call attention to some facts which may be helpful in securing true models.

An impression which has been secured by means of an improper material or the excessive use of a proper material is not to be depended upon. These are likely to have distorted the tissues so that a model made from them does not represent the mouth in its normal repose. An impression made with an improperly fitted tray is likely to be inaccurate. In practice I often, when unable to secure a desirable impression by the use of trays in my possession, get the best model I can and from it make metal dies and swage from suitable sheet metal a tray which will approximately conform to the case in hand. In such a tray, by the use of a limited quantity of suitable material, I am able to secure an accurate impression with little distortion of the soft structures. The impression is the matrix in which the model is to be cast, and unless it is correct the model will not represent the actual conditions of the mouth. Every feature of the mouth must be counterfeited in the plaster model so that it may be transferred to the zinc die.

All subsequent proceedings are of equal importance. The plaster must be properly prepared for pouring. The impression must be properly prepared to receive the batter and to separate from it after hardening has occurred. The impression material used will determine the medium used for favoring separation of the impression from the model. If the impression be of wax or modeling compound, it is only necessary to wet it. If it be of plaster of Paris, it may be soaked for a few minutes in water previous to pouring into it the plaster batter. Sometimes it may be necessary to treat it to a coat of shellac varnish, and then to some oily substance.

The size and shape of the plaster model will vary with the use to be made of it and the character of the impression. As a rule, small models should be poured first and the plaster built up over the palatal arch so that the model will be uniform as to thickness ; this will insure more uniform contraction of the plaster while setting and the vault of the arch will not be distorted by the unequal contraction in the thicker parts of the model. After the small model has been separated from the impression it may be built up to any desired size by the addition of soft plaster and reversed on a glass slab to get a symmetrical model which will set level in the moulding flask. This also gives opportunity to tip the model so as to give it what moulders call "draft;" that is, a chance to withdraw a model containing considerable undercut from the sand without distorting the sand matrix. Care must be exercised in this matter that the model be not thrown out of plumb to such an extent as to render it impracticable to use a die made from it with any degree of certainty. For the reason that it is difficult to strike an unsymmetrical die with a swaging hammer directly over its equilibrium, and at the same time drive the plate in the proper direction. Usually it is possible to give the model considerable bevel or draft at the posterior part. This assists in withdrawing the anterior alveolar ridge from the sand without changing its shape. This beveling of the model should not be indulged to sufficient extent to permit rocking or tipping of the die while swaging.

The size of the model will depend upon the size of the metal die required. Usually a die should be only so high as to insure stability and convenience in handling. But sometimes a larger die is necessary to overcome special difficulties in adapting plate to irregular dies, or in utilizing thick plate. A high die can be made where a low model has been used to get the matrix by placing a cast iron moulding ring on the top of the flask immediately after pouring the zinc into the mould, and then, before the zinc has crystalized, add to its height by pouring this ring full of melted zinc. It is a good idea to use a shallow die so that the melted die metal shall not have so far to fall as in a deeper mould, hence lessening the possibility of displacing the sand by pouring the heavy metal from too great a height. The beveled slope on the posterior part of the model here serves a good purpose. Instead of pouring the zinc directly into the mould it can be directed from the mouth of the ladle down this incline, so that it does not disturb the delicate lines of the sand matrix at all. After the matrix is poured level full then the small cast ring is adjusted to secure the needed depth to the die. This is an advantage as it gives the metal already poured time to chill and so prevent its perforation by the gasses formed from the hot metal coming in contact with the wet sand. It also extends the skrinkage into the upper part of the die where it does not matter whether this part of the die corresponds to the model or not.

The preparation of plaster models so that they will readily draw from the sand is important. But the treatment of the surface of the model is of the utmost importance. Usually models are best trimmed to form as soon as the plaster has set, but they should never be varnished until thoroughly dry. A model that is varnished while it is still green will not pull well from the sand; it will preserve sufficient moisture to cause the sand to adhere to it, and so mar the sand mould when it is removed. A green model will not only not absorb the varnish, but it requires thicker varnish and more coats to get a smooth surface. This is, of course, objectionable, because it distorts the surface of the model and obliterates the sharp lines.

A model should be thoroughly dry before any varnish at all is applied. And this drying can not be facilitated usually by artificial means. Never try to dry a model on gas stove or Bunsen burner, as this will surely destroy the integrity of the model and render it impossible to secure a smooth surface. If possible let the model stand several days in a moderately warm and dry room, then it will take the varnish readily. The first coat of varnish should be rather thin, and when applied to a dry model it ought to penetrate nearly an eighth of an inch. A second or third coat applied with sufficient intervals for hardening will produce a smooth and impervious surface which will not favor the adhesion of the sand, and one that will readily pull from the sand mould.

The varnish should be applied with a large brush, and quickly, so as to prevent solution of preceding coats, which will give a roughened surface. Sufficient time should elapse between varnishings to allow the several coats to become hard.

The making of models for seamless crowns, etc., is another subject, and I will not discuss it, although I have with me some specimens of models used for this purpose.

